# Physical Activity during the School Holidays: Parent Perceptions and Practical Considerations

**DOI:** 10.3390/ijerph16101697

**Published:** 2019-05-14

**Authors:** Lydia G. Emm-Collison, Sarah Lewis, Thomas Reid, Joe Matthews, Simon J. Sebire, Janice L. Thompson, Russell Jago

**Affiliations:** 1 Centre for Exercise, Nutrition & Health Sciences, School for Policy Studies, University of Bristol, 8 Priory Road, Bristol, BS8 1TZ, UK; s.c.lewis2010@gmail.com (S.L.); tom.reid@bristol.ac.uk (T.R.); joe.matthews@bristol.ac.uk (J.M.); simon.sebire@bristol.ac.uk (S.J.S.); russ.jago@bristol.ac.uk (R.J.); 2 School of Sport, Exercise and Rehabilitation Sciences, University of Birmingham, Birmingham, B15 2TT, UK; J.Thompson.1@bham.ac.uk

**Keywords:** physical activity, children, school holidays, holiday clubs

## Abstract

Children’s physical activity decreases during school holidays. Less structured days and reduced participation in organised activities may account for some of the decrease. Little is known about the factors that influence parents’ decision to enrol their child in organised activity such as holiday clubs. This paper sought to explore parents’ perceptions of their child’s physical activity during school holidays and the factors that influence holiday activity-based decision making. Semi-structured telephone interviews were conducted with 42 parents of children aged 10–11 years in July 2017 or March 2018. Data were analysed using a combination of inductive and deductive content analysis to explore parents’ perceptions of holiday-based physical activity and the factors associated with how they provide physical activity opportunities for their children. The data revealed that most parents consider their child’s physical activity levels when planning for the school holidays. However, work commitments in the holidays meant many parents had to rely on both informal and formal childcare. Grandparents were the primary source of informal childcare, despite a perception that children were not as physically active when with them. Holiday clubs were also a viable option, but the cost, location and age-appropriateness of provision inhibit parents signing older children up to these regularly.

## 1. Introduction

Physical activity is associated with a reduced risk of disease, including heart disease, stroke, type 2 diabetes, several forms of cancer, and depression [1,2]. It is recommended that children engage in an average of 60 minutes of moderate-to-vigorous physical activity per day [3,4,5] and activities which strengthen their muscles and bones three times per week, but evidence suggests that most children do not meet these recommendations [6]. Most physical activity interventions have focused on the school day, with several national strategies encouraging more physical activity during term time (e.g., The Daily Mile [7], Active 30:30 [8]). Some school-environment based programs have been shown to have beneficial effects on children’s physical activity during the school term [7], but there is little consideration of physical activity during the school holidays. 

There is consistent evidence showing that children’s physical activity levels decrease during school holidays and are particularly low at the point of returning to school following longer holidays [9]. Whilst shorter holidays appear to have little influence on physical activity behaviours, after a 3-week holiday, children engage in 33 minutes more sedentary behaviour and 12 minutes less moderate-to-vigorous physical activity (MVPA) per day [9]. Further evidence has also shown differences between school term and school holiday behaviours in primary-school aged children [10], with higher sedentary behaviour and decreased light activity during holidays. There is also some evidence of increases in the prevalence of overweight and obesity seen after holidays [11,12]. The structured days hypothesis suggests that obesity-related behaviours (including physical activity, sedentary behaviour and diet) are beneficially regulated during more structured days (e.g., a school day) than on less structured days (e.g., a school holiday day) [13]. Evidence supports this hypothesis, indicating that physical activity levels are lower on weekend days compared to weekdays, particularly for less active children [14]. Increasing PA during school holidays, and outside of school time more generally, has been identified as a target for health interventions to try and decrease the prevalence of unhealthy weight gain over this time [9]. Evidence suggests that the differences between term-time and holiday-time activity levels could be attributed to participation in organised physical activity, with greater decreases in activity observed in children who do not participate in organised activities during the holidays [11]. The limited evidence of the physical activity acquired whilst at a holiday club indicates they may also help children to meet the recommended levels of MVPA [15]. Evidence from intervention studies suggests holiday clubs show promise for increasing holiday physical activity levels in primary-school age children [16,17] and may have particular benefits for girls [17]. Additionally, organised activities during the school holidays may be particularly beneficial for young people’s mental health and well-being [18]. Collectively, this indicates that increasing participation in more structured activities during the school holidays could be have significant public health benefits.

Little is known about parents’ decision-making process in relation to how holidays are spent, or the factors that influence parent’s decisions to enrol their children in different holiday activities. Given that primary-school aged children rely on parents to provide logistical support related to their physical activity (e.g., paying fees, buying equipment or clothing, transport) [19], it is important to understand parents’ perspectives on current holiday activity provision and the factors that influence their decisions to engage, or not, with organised holiday activities. With these gaps in the literature in mind, using qualitative interviews with parents of year 6 children, the current paper explores: (1) Parent perceptions of their child’s physical activity in the school holiday compared with term time; (2) the use of different childcare options during the school holidays; and (3) the factors involved in decision-making regarding holiday activities.

## 2. Materials and Methods

This study used data from the B-Proact1v cohort study, which has been described in detail elsewhere [20,21,22]. In brief, the study aimed to explore the factors associated with children’s physical activity, sedentary time and screen-viewing throughout primary school. Data collection occurred in three phases: In 2012–2013, when children were aged 5–6 years; in 2015–2016, when the same children were 8–9 years; and in 2017–2018, when the same children were 10–11 years. In the first phase, 1299 children and at least one parent from 57 primary schools were involved, and the same 57 schools were invited to participate in phases 2 and 3. Forty-seven schools and 1223 child–parent dyads participated in phase 2, and 50 schools and 1296 children–parent dyads participated in the third phase. The present study uses participants involved in the third phase. Children and parents wore an ActiGraph wGT3X-BT accelerometer mounted on their waist for five days, only removing the device for water-based activity (e.g., bathing) and sleep. These data were processed using Kinesoft (v3.3.75; Kinesoft, Saskatchewan, Canada), and participants were only eligible for inclusion in subsequent analyses if at least three days of valid data (including at least one weekend day) from both the child and parent were provided. Valid days were defined as at least 500 minutes of data after excluding intervals of ≥60 minutes of zero counts, allowing up to two minutes of non-wear time. Minutes spent in moderate-to-vigorous-intensity physical activity (MVPA) were derived using population-specific cut points for children [23] and adults [24]. Children’s height, weight and blood pressure were also measured.

**Figure 1:**
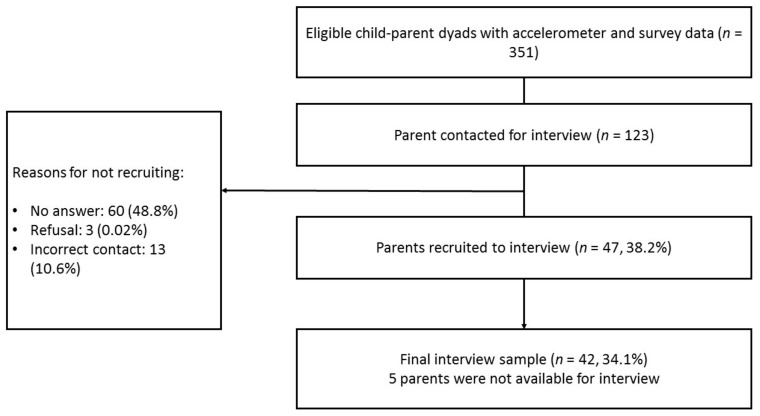
Study flow of participants.

Semi-structured telephone interviews were conducted with a sub-sample of 42 parents in July 2017 (20 parents) and March 2018 (22 parents). Telephone interviews were chosen as the method due to the flexibility they offer both participants and researchers and because they are a cost-effective way of collection information from a range of people [25]. Participants were sampled based on the child’s accelerometer-estimated MVPA minutes per day. Eligible participants were stratified into three groups of low (<51.0 mins/day), mid (51.0–71.8 mins/day) and high MVPA (>71.8 mins/day) and further split by child gender. To ensure that the groups were distinct, only those in the low and high MVPA groups were eligible for the interview sample (*n* = 351), giving four groups (low MVPA boys, low MVPA girls, high MVPA boys, high MVPA girls). The order in which parents were contacted to participate was randomised within each group by an independent researcher. A total of 123 parents were contacted, 47 (38.2%) agreed to participate and 42 (34.1%) completed an interview (Figure 1). Interviews were audio-recorded using an encrypted digital voice recorder (Olympus DS-3500), and recruitment continued until theoretical data saturation had been met, identified through the repetition of themes. Interviews were conducted at the participant’s convenience, and all participants provided recorded verbal consent at the start of the interview as per the ethical agreement. As a thank you for their time, all participants received a £10 high street gift voucher following the interview. Ethical approval for the study was granted by the School for Policy Studies Research Ethics Committee. 

### 2.1. Interview Data

A semi-structured interview guide was developed and refined iteratively by the research team. The guide was informed by gaps in current knowledge and guided by previous research. The interview was concerned with understanding parents’ perceptions of child physical activity during the school holidays. Specific topics that were explored include parents’ perceptions of their child’s physical activity during school holidays, the differences between child physical activity during term time and school holidays and the factors that influence physical activity during the school holidays. Example questions included ‘What factors influence your decision making/plans [during the school holidays]?’, ‘Does [child] attend any new or different clubs over the school holidays?’, and ‘Do you consider the amount of time [child] will spend being active when making plans for the school holidays? If so, why?’. To explore a range of experiences, approximately half of parents were interviewed in July to reflect on the upcoming summer holidays, and half were interviewed in March to focus on the upcoming Easter holidays. To allow participants to shape the direction of the interview, questions were posed in a non-leading manner, and interesting responses were probed with further questions. Two male researchers trained in qualitative methods conducted the interviews (JM, TR), and the researchers met regularly throughout data collection to discuss the interview guide and make minor changes. Researchers’ interpretations of responses were checked for accuracy during the interviews. At the end of the interview, key findings were summarised, and participants were given the opportunity to comment further on any of the topics discussed at the end of the interview.

### 2.2. Demographics

As part of the wider study, parents completed a questionnaire online (*n* = 25) or on paper (*n* = 17), providing parent and child gender, date of birth and parental employment status. An index of multiple deprivation (IMD) score was assigned to each child based on their home postcode as an indicator of socioeconomic status. The scores are based upon the English indices of deprivation [26], with higher scores indicating higher levels of deprivation.

### 2.3. Data Analysis

Interviews were transcribed verbatim. All transcripts were checked for accuracy and anonymised prior to being entered into QSR NVivo 11 (QSR International, Warrington UK) for analysis. Interview data were analysed using the framework method [27], which allowed us to take a combined approach to analysis, enabling themes to be developed both inductively from the accounts of research participants, and deductively from existing literature. This method involved several stages of analysis: Familiarisation, coding, framework development, framework application, and interpretation. During familiarisation, three researchers (LEC, SL, TR) independently read and re-read all interview transcripts and listened to audio-recordings to immerse themselves in the dataset. After familiarisation, each researcher independently read a sub-sample of three transcripts line-by-line and provided annotations to describe key passages of text. The researchers then discussed the annotations and collaboratively developed an initial coding frame with the principle investigator (RJ) to apply to subsequent transcripts. This process was repeated for a further three transcripts, and the framework was refined. This process occurred until no further refinements were required, and the researchers met regularly to ensure consistency in the application of the framework. Any disagreements in coding were discussed with additional members of the team (RJ, SS) to come to a collective consensus. Hierarchies of themes were created, and summaries with accompanying quotes for each category were extracted to aid reporting. Participant characteristics are presented using means and proportions. Further, to contextualise the quotes, they are presented alongside the parent’s relationship to the child, child gender and MVPA classification.

## 3. Results

Table 1 shows participant characteristics. Twenty-one participants were mothers and 21 were fathers, with an average age of 42.5 years (30.5 to 53.4 years). The sample was predominantly white British (95.2%), with the remaining sample being white ‘other’. The mean IMD score overall was 16.9 [26], and IMD was higher (indicating more deprivation) in the low activity groups than the high activity groups. Mean child MVPA overall was 64.6 mins/day, with distinct differences in the low and high activity groups by design. Mean parent MVPA was 58.9 mins/day, with similar patterns between low and high activity groups. Most parents worked either full-time or part-time (90% in total). A total of 34 parents interviewed were married or cohabiting with the other parent, and 7 were separated from the year 6 child’s other parent. Seventeen of the children had at least one older sibling, 17 had at least one younger sibling and 2 were only children. One family had grandparents living at the same address. 

Parents were asked about their perceptions of their child’s physical activity during the school holidays compared to term time, and 18 felt their child did more activity during the holidays, 13 felt their child did less activity during the holidays and 10 felt their child’s activity levels were roughly the same. Primary reasons for their child engaging in more activity were the greater proportion of free time, longer days, and opportunity to engage in activities for a longer period of time (e.g., at sports or holiday clubs). Where parents felt their child engaged in less activity, the main reasons were due to a reduction in active travel, the lack of PE and the discontinuation of organised activities during the holidays. 

Four broad themes emerged: Parents’ consideration of physical activity during the school holidays; when real life kicks in; childcare options during the school holidays; and practical considerations.

### 3.1. Parents’ Consideration of Physical Activity during the School Holidays

The majority of parents reported that keeping their child physically active was a consideration when organising school holiday activities (*n* = 28). For many parents, the main reason for considering physical activity was to balance time spent active against time spent sedentary, mainly due to strong child preferences for screen-viewing:

“So the idea is for him to be involved in exercise and having time with his friends because he likes the X-Box and would live on it if he had the chance.” (Interview 10, Mother of High PA boy).

“We do, yeah. Er, we don’t want him just sitting inside watching telly all holiday, so er, yeah, there’s er, there’s not going to be, not going to be lots of time for that.” (Interview 19, Father of Low PA boy).

“Yeah cause she’s got a tendency to sit down on her iPad or her iPhone and watch like YouTube or chat to her friends on it, you know what I mean?” (Interview 2, Father of Low PA girl).

For parents who did not explicitly consider physical activity during the holidays, it was generally due to perceiving their child as being active already, due to their preference for being outside and moving:

“When it comes to summer, and she is a child that um, prefers to be out, she prefers to be social, she prefers to be active rather than sat down watching telly. She’s not really one um, she’s kind of a polar opposite of her brother really um, in some respects um, so she’s not happy being sat in all day doing nothing or watching telly.” (Interview 14, Mother of Low PA girl).

“Erm that’s not one of our primary concerns cos the holidays that we do are generally relatively active anyway so it’s not something, we don’t think we need to do something because we need to be active we do stuff cos we’ll be active doing it anyway.” (Interview 1, Father of High PA boy).

“She’s really not the child that I need to boot off the sofa at all unlike her sister.” (Interview 25, Mother of High PA girl).

Some parents said that they only consciously consider their child’s activity when they notice that they are not being active:

“No—no I don’t think—I don’t think we consciously think about you know, ‘oh we need to make sure she’s active and she’s doing things’. There is a sort of unconscious realisation that if during the holiday she’s becoming particularly inactive that we will encourage her to do stuff like go out in the street and play with her friends you know to get her out and about and actually not sort of being lethargic.” (Interview 27, Father of High PA girl).

Most parents recognised the importance of keeping their child active during the holidays, with the majority referring to the health benefits of maintaining an active lifestyle (e.g., ‘general health’, ‘because it’s healthy for him’, ‘weight’ and ‘the benefits emotionally’). However, for many parents, the logistics of their child being active and how it made elements of their day easier (e.g., managing behaviour, promoting good sleep) or more difficult was the most influential factor in their support for physical activity:

“More yeah if they’re not active then they’ll all start arguing over little things whereas if they’re kept active and doing stuff they’re focused on that and they’re not at each other quite so much.” (Interview 3, Mother of Low PA girl).

“Yes because the more active she is, the more she’ll sleep.” (Interview 6, Mother of High PA girl).

“Because if the weather is like really bad and they haven’t been able to get out, like I sort of said, and you know, kids do go stir crazy.” (Interview 8, Mother of High PA girl).

Most parents valued maximising their child’s enjoyment of the school holidays by allowing them time to relax, engage in their preferred activities and create positive memories through shared experiences. Generally, making friends, developing skills and being outdoors were seen to be enjoyable aspects of physical activities: 

“They love it [holiday club]. They absolutely love it and they have met so many friends which is good and that’s why they like going back every time.” (Interview 34, Mother of High PA boy). 

“She just can’t stand, she gets bored easily, so she likes to be out and about and doing things rather than sitting at home, if I suggest a duvet day, she’s like, she’s up for it for an hour and then she’s bored…”(Interview 16, Mother of High PA girl).

“So erm, the Snowden trip, for example, we, we know—we’ve talked to them about that. We know they’ll, they’ll both like that, so [his brother] is going to go on the train; he’s keen to try and walk up Snowden” (Interview 19, Father of Low PA boy).

### 3.2. When Real Life Kicks In

For many parents, despite recognising the benefits of being active, balancing working and childcare during holidays had an impact on child physical activity. Parents commonly discussed not being able to facilitate physical activity and that their children engaged in more home-based activities, often more sedentary in nature, particularly when parents were working from home. 

“On a Monday and a Friday he’ll be at home with me because I work from home. Erm he doesn’t get up to a lot on those days at all, he just sits and plays on his games.” (Interview 22, Mother of Low PA boy).

“Yes both my wife and I work and, er, obviously if we’re not available, er, then we’re unable to take them places and enable things.” (Interview 38, Father of High PA boy).

“Because there are days when just real-life kicks in and it’s like just play, play in the garden, you know, and amuse yourselves all day, um, if I’m working. But, um, we do, we do try to counteract that as well, sometimes if it’s got in to a bad habit.” (Interview 37, Mother of Low PA girl).

However, for one working parent, the rarity of days off together in the school holidays meant these days are used as an opportunity to make the most of time together:

“Yeah, but when I’ve have them like in the half terms and things like that, I’ve said, come on, we’ve got to go out and do something, I don’t want her sitting in, I like to make the most of it, do you know what I mean? Make the most of the time I’ve got.” (Interview 13, Father of High PA girl). 

#### 3.2.1. Childcare Options during the School Holidays

In line with the majority of the parents needing to continue with work during the holidays, many parents used various informal and formal forms of childcare, such as holiday clubs and support from grandparents.

##### Grandparents as a Source of Childcare

For many families, grandparents offered one of the primary sources of childcare 

“Yeah, I work shifts so it can, it depends on what day of the week it is I can be off with him if not he goes to his grandparents’ house primarily.” (Interview 1, Father of High PA boy).

“Okay um, the majority of the holidays, to be honest, I will be working so she will be in the care of her grandparents um, on a daily basis she’ll be there for about five/six hours um, four days a week.” (Interview 14, Mother of Low PA girl).

Whilst a large proportion of the children were going to be looked after by grandparents at some point during the holidays (40%), parents were largely unaware of the specific activities their child would be doing whilst with the grandparents, suggesting that parents are not prescriptive in their child’s activities when not in their direct care. Nevertheless, parents assumed time with grandparents would involve a mix of housework and gardening, day trips and sedentary activity: 

“ You know, he does a bit of gardening with his grandma as well. So, um, yeah.” (Interview 15, Father of Low PA boy). 

“But they usually take him out, they don’t—he can’t go on his Xbox or anything like that, erm so they take him out.” (Interview 5, Father of High PA girl). 

“I think there are, should be then round grandparents I believe as well so again there might be a mixture of, erm, indoor electronic activities and round the park hopefully.” (Interview 5, Father of High PA girl).

One parent also explicitly highlighted that their child has less autonomy over their activities when under the care of grandparents: 

“Nan might not be able to go anywhere or, ‘You’re going round nan’s’, and nan might have said, ‘Right, we’re going to go down to, into Bristol and go round the, the docks’, or that sort of thing. So the girls probably wouldn’t have much input on that.” (Interview 5, Father of High PA girl).

##### Holiday Clubs

Parents of children who attended holiday clubs generally chose the club due to the range of activities that they offered and how these activities align with their child’s needs and preferences: 

“So er, he goes to er, like a, a place called Shine erm, which is a, a sort of sporty activity holiday camp and—er, well, Holiday Club er, and also he goes to er, [council] Leisure Centre which run a, a Holiday Club erm, in the school holidays as well. Erm, so it’s, it’s a mixture; sort of some sporty stuff and activity stuff. They do a bit of all sorts with them at, at both of those clubs.” (Interview 19, Father of Low PA boy). 

“Just because he’s got ADHD and so he needs a good run around now and again. Erm also because I don’t agree with these holiday clubs that just sit in a room all day erm and watch TV or just playing painting or something. That’s why I’ve got him in this certain one, it’s because they do a lot of activity.” (Interview 22, Mother of Low PA boy). 

“Yep so that’s based, erm, at their, er, at the site of their primary school. Erm, they currently go there for breakfast some days and, erm, so they will be doing…they will be there for the day and will do various activities throughout the day both indoors and outdoors.” (Interview 29, Father of High PA boy).

A few parents indicated that their child’s preferences have changed as they have got older, and one queried whether her son was getting too old for his previous holiday club provision:

“Yeah I mean we have looked, we have had to look to sort of change what we are doing on the summer holidays erm because quite a lot of the activities he used to do no longer are of interest.” (Interview 9, Father of High PA boy).

“In February he went to a football, dodgeball, I don’t know, all sorts of sports things down local to us. He did that one day but that was only one day. Erm I don’t know if he’ll want to do it this time. I don’t know if he’s getting a bit old. But if there was a football one or—for his age, then he would—I think he would happily….” (Interview 30, Mother of High PA boy).

Whilst some parents expressed that their child enjoyed the holiday club they attend, across all the parents, views on holidays clubs were mixed. Some parents described previous experiences with holiday clubs as being very negative, with one parent portraying holiday clubs as ‘soulless’: 

“Um, one of the reasons for getting him involved and doing different things and for me to be home is so that he didn’t necessarily have to go to um, just to general holiday clubs because sometimes they seem a bit soulless and if he hasn’t—yeah, he hasn’t got—he hasn’t got a friend there anymore, it’s not—you know, he comes out a little bit um, ‘it wasn’t that good, I didn’t know anyone, I didn’t have anyone to play with’ so—and this year I’ve tried to not have to put him clubs where he’s not going, you know, with somebody so…” (Interview 10, Mother of High PA boy).

This highlights the importance, for some children, of having friends at holidays clubs and facilitating participation in more structured activities, as well as the aforementioned free-play. An issue that parents frequently raised was the fact that a lot of physical activity clubs were only provided in school term time and stopped in the school holidays, which had a potentially negative impact on the amount of PA their child undertook. For example: 

“I mean, well he would be playing football now because he belongs to a football club but that’s during the Easter holidays that doesn’t happen“ (Interview 38, Father of High PA boy).

“He does a lot of after school activities at the moment but obviously that is going to stop in the next couple of weeks because of the summer holidays so he will find himself with a bit more time on his hand“ (Interview 18, Mother of Low PA boy).

However, several parents identified that their child’s activity was supplemented by more free play and family activities, such as playing on a trampoline, skipping, walking, and bike rides. 

“So I would look to be doing something physical with them, we still continue with their swimming lessons during the, [yeah] during the summer, during the school holidays but the dancing stops [ok] so that’s term time only so we sort of replace that with other things, erm, we don’t engage in any sort of childcare or holiday camps or anything like that because we don’t need to, we do it as a family.“ (Interview 12, Father of Low PA girl).

### 3.3. Practical Considerations

In addition to work commitments, there were several more general factors that influenced the activities that children engaged in during the school holidays. These included child enjoyment, cost and location, of which are important practical considerations for service deliverers. 

#### 3.3.1. Financial Concerns

Finances and the cost of activities was pertinent throughout the interviews but were particularly salient in decisions regarding childcare options. Specifically, parents cited being unable to send their child to the holiday club they wanted due to the cost:

“He doesn’t do a holiday club generally, but I’m sure he would like to. If I said… I’m sure he would like to be at a football camp or something like that or if there was a rugby camp, erm. But yeah, he would probably be up for that but it’s just being able to afford it all.” (Interview 15, Father of Low PA boy). 

“It can be in terms of you know what we are able to do yeah because, er, if activities are costing, er, then that is a big disincentive say for example what she’s doing at the moment has a very low cost, she doesn’t have to pay to do it, er, you know so it’s just the cost of getting her there and back. So that works well but, er, things like you know there’s sort of holiday clubs that children go to, er, that really you need, we wouldn’t do that, we might do it as a one off, there’s an opportunity for her to do gymnastics, er, but rarely do we take those up just because obviously it would cost a lot of money if we were doing it on a regular basis during the holidays.” (Interview 27, Father of High PA girl).

“But obviously we can’t do everything, can’t afford everything [yeah] and so that’s it really” (Interview 15, Father of Low PA boy). 

One parent discussed how they have developed creative alternatives to organised holiday clubs that still enable their child to do the activities they want without the financial implications, feeling that this was particularly possible due to them living in an urban environment:

“We’re … in the city, you don’t actually need to spend money, we’re quite creative with our thinking. So, I wouldn’t say, it just makes me think sometimes, oh, I wish we could do a bit more or we could do, you know, like, a tennis club or something? I’m keeping it sporty for the purpose of the interview but it’s like, actually, no, we’ll just have to take our own rackets and ball down to the park if we want to do that, um, yeah, that’s just, that’s just how it is, it’s ok. We’re not, we’re not, I say we’re a bit skint, we’re not on the poverty line, you know, it’s not that bad, we just have to be a bit sensible right now.” (Interview 37, Mother of Low PA girl).

#### 3.3.2. Accessibility of Provision

Parents expressed a need to manage their child’s expectations of what is possible in the holidays, particularly with regards to location of home and of facilities. For example: 

“It’s a bit difficult here because we live in the middle of nowhere, so you know there’s nowhere really for them to go, er, out and play, you know they don’t have any friends that are here, er, because we live quite far away from their school and their mother so.” (Interview 33, Father of Low PA girl).

“I mean he would probably like to be more active but he needs parents to sort of take him to a football pitch or you know certainly he has needed that and we’re not always available to do that.” (Interview 38, Father of High PA boy). 

Location was a logistical problem for families from more rural areas. This meant that parents had to be realistic about the time to travel to facilities, particularly noting that facilities or activities that were far away could not occur daily, as parents simply did not have the time, energy or money to maintain a constant level of PA: 

“I think you’d probably struggle to find any more time. Yeah that’s the issue we just as I say there’s always something going on in our house so time is of a real premium erm but he does, I mean in a normal week he does three hours of tennis on varying days.” (Interview 1, Father of High PA boy). 

“Oh, I, I don’t think there’s a main one. It’s me and my wife together erm, but, but we kind of know what the kids like, so—erm, but, you know, there’s certain amount of er, things that have to be done, like er, going to see erm, you know, recuperating grandparents.” (Interview 19, Father of Low PA boy).

Parents also expressed concerned regarding the breadth of provision (including fewer options for older compared to younger children) and limited availability within/high demand for clubs: 

“We are hoping that he’s gonna be going to erm summer holiday club, which he normally does do, but it’s just whether or not he gets a space.” (Interview 22, Mother of Low PA boy).

“There’s not really a lot of like, when I was younger there was like, things like play schemes like, and there’s not, I don’t think there’s any of them actually.” (Interview 16, Mother of High PA girl). 

## 4. Discussion

The data in this paper provide new evidence for parents’ views on the opportunities and challenges in keeping children physically active during the school holidays. Some parents perceived their child’s physical activity to increase in the holidays, whereas others felt that it decreased or did not change. In line with the structured day hypothesis [13], the reasons given were largely related to the structure of the day, with some suggesting the lack of structure was positive (e.g., longer days meant more free time) and others suggesting it was negative (e.g., organised sports clubs stopping during holidays). In most cases, parents who perceived their child’s activity to be higher during the school holidays than term time attributed this to them having more time and opportunity to be involved in organised activities such as holiday clubs and sports camps. Similarly, the majority of parents said they did consider their child’s physical activity levels when making decisions regarding school holidays. It was a particularly important consideration for parents whose child preferred screen viewing to being active, with parents wanting to ensure this screen viewing was balanced with activity. Previous research has highlighted the idea of ‘digital balance’ as an important family consideration during school term time [28], and our findings indicate that, for many, this concern translates to holidays. However, physical activity being a consideration for many, there were more practical considerations, such as work commitments and cost, which inhibited parents’ abilities to ensure their child engaged in sufficient activity every day.

The majority of parents in our sample were in part- or full-time employment, and our interviews revealed the balancing act that parents undertake during school holidays and the potential influence this has on child physical activity. The proportion of working mothers has increased over the last decade, with an estimated 74% of mothers working full- or part-time in 2018 [29]. A total of 72.5% of two-parent households had both adults in some form of employment, and almost half (45.5%) had both parents in full-time employment [29]. In our interviews, parents recognised that for at least some of the holiday days, the need to work took precedence over keeping their child active and busy. Particularly on days when working from home was required, parents acknowledged that they were unable to facilitate their child being active, and therefore, their child would default to being sedentary and engaging in screen-viewing. This highlights that, for the many parents in the UK, school holidays involve balancing parenting and work duties, and therefore, some form of childcare is a necessity for many families. More opportunities for children to be active close to home, such as through drop-in sessions in parks and community spaces, may offer a feasible way for children to remain physically active without reliance on parent’s for logistical support. 

Parents’ working arrangements led to many families using some form of childcare, commonly grandparents. Grandparents are a regular source of childcare both in and out of school holiday time due to the convenience and free or low cost compared to other options [30]. Evidence indicates that informal childcare options, such as grandparent care, are associated with higher body weight in younger children [31], and there is some evidence indicating that time with grandparents may reduce older children’s activity levels [32]. Parents were generally unaware of the activities that their child did whilst in the care of grandparents, although broadly, parents assumed it would involve a combination of sedentary activities and day trips. This lack of awareness is consistent with previous research showing that parents and grandparents rarely discuss their child’s physical activity due to the inherent trust in grandparent care [33]. Given the prevalence of informal childcare options such as grandparents, particularly during the holidays, there needs to be additional work to further identify the key patterns of activity and sedentary behaviour when under the care of a grandparent to help to develop targeted interventions. Additionally, and given that previous research has suggested that grandparents’ own access to and engagement with PA is a key influence on how active the child is when under their care [33], identifying ways to support grandparents in remaining active themselves and with their grandchild may have significant public health benefits for young people and their grandparents [34].

Several parents used holiday clubs as a source of childcare, and attendance at a club or sports camp was one of the key reasons for parents perceiving their child’s physical activity levels to increase during the holidays. Similarly, the cessation of organised activities provided in term-time during the holidays was a primary reason for perceptions of decreased activity. This aligns with the structured days hypothesis [13], indicating that days structured around organised activities, such as holiday clubs or sports training, inherently encourage more physical activity. Child participation in organised sports during term time has been shown to be associated with higher levels of MVPA [35]. Additionally, existing intervention studies have shown that holiday clubs do have the potential to increase PA [17,36]. However, this effect is not consistent and may only be applicable to boys [17]. More work is needed to ensure consistency in the quality of holiday club provision with a particular focus on engaging less active children (e.g., girls). 

Parents expressed concern regarding whether their children would be able to get a place in the holidays clubs that they wanted to attend, indicating there may be issues with the levels of provision. Recent statistics suggest that only 25% of local authorities in England provide enough holiday childcare for working parents [37]. Related to this, several parents expressed concern over the suitability of the clubs on offer for their child, citing changing preferences as a key reason why clubs their child used to enjoy are no longer age-appropriate. The Holiday club survey [38] indicated that the majority of UK-based holiday club provision is focused on primary-school aged children (i.e., 5–11 year olds). Holiday clubs provision reduces as children get older and, by the time children are 12–14 years, only 14% of local authorities offer enough holiday clubs, with none of the local authorities in the South West of England (where the present study was conducted) meeting demand [37]. Our data also indicate that fewer clubs are provided during the Easter holidays when compared to summer holidays [37]. Therefore, there needs to be an increase in the level of holiday club provision that is targeted at older children (11+) and that is on offer consistently at different times of the year. 

Many parents highlighted that their child had expressed an interest in attending a club but that they are unable to allow their child to attend due to the cost of the activities. A recent survey of holiday childcare in the UK reported that, in 2018, a week of childcare during the school holidays costs £133, an increase of 4% from 2017 [37] which is higher than the average salary increase for the same period (3.1%). Therefore, formal childcare options, such as holiday clubs, are becoming a less viable option, particularly for low-income families. However, given recent statistics suggesting that many holiday clubs in the UK are free of charge [38], there may be a need for better signposting for parents towards free or low-cost options.

Location was another key factor when deciding upon holiday activities, and this issue was brought up primarily by parents from more rural areas. Whilst provision of sufficient holiday childcare nationally is already low, with only a quarter of local authorities offering sufficient levels of childcare options, the levels are dramatically lower for rural communities at 9% [37]. Future strategies targeting holiday-based physical activity should consider both the location of cost of provision to ensure that poorly-resourced groups are not excluded. In Wales, there has been success in running physical activity-focused holiday clubs on school-sites [36], which might offer a low-cost and accessible option for many rural communities. The interviews suggest that, for parents, practicalities such as cost, location and age-appropriateness are more pertinent in decision-making than whether a childcare option will provide sufficient opportunities for physical activity. Therefore, holiday-club based interventions aiming to increase children’s physical activity should also aim to minimise these common barriers to parental engagement. Table 2 summarises the key findings and the related recommendations for strategies to support children’s physical activity during the school holidays. 

### Strengths and Limitations

The present study has several methodological strengths, including the recruitment of a relatively large sample of parents from a diverse range of socioeconomic backgrounds and with children whose PA levels spanned low to high. Additionally, half the sample of parents are fathers, a group who are traditionally difficult to recruit to research projects [39]. The study is, however, limited, as it relies on parental perceptions of their child’s behaviour during the school holidays, and we do not have the accelerometer data to ascertain how parents’ perceptions match reality. The sample were recruited from a single area in the UK, and therefore, the parents’ experiences of holiday activities might not be representative of the UK population, particularly given the disparities in holiday care provision across the UK [37]. Finally, the sample were largely in full- or part-time employment, and so, considerations around holiday activities and childcare may be not be consistent across parents who do not work. Gaining the perspectives of more non-working parents would be useful for informing future behaviour change strategies.

## 5. Conclusions

Children’s physical activity decreases during school holidays, emphasising a need for strategies to promote more holiday physical activity. This paper presents the first exploration of parents’ perceptions of their child’s physical activity during the holidays and factors that inform decision making with regards to holiday activities. Whilst most parents recognise the importance of physical activity for their child and many consider it when making holiday decisions, the reality of having to work means many parents do not have the capacity to provide daily opportunities for their child to be sufficiently active during the school holidays. The primary sources of childcare during the holidays are grandparents and holiday clubs, and therefore, more support for grandparents in terms of providing physical activity opportunities for children when in their care is needed. Additionally, holiday clubs that are more affordable, targeted at older children and run in a variety of localities are required. 

## Figures and Tables

**Table 1:** Characteristics of the sample of parents (*n* = 42) and their children by group and overall.

	High PA Boy (N = 10)	Low PA Boy (N = 11)	High PA Girl (N = 10)	Low PA Girl (N = 11)	Overall (N = 42)
	Mean (SD)/%	Mean (SD)/%	Mean (SD)/%	Mean (SD)/%	Mean (SD)/%
**Child characteristics**					
Gender (% female)	-	-	-	-	50.0%
Age (years)	10.7 (0.5)	10.8 (0.4)	10.9 (0.3)	10.8 (0.3)	10.8 (0.4)
BMI z-score(kg/m2)	0.1 (0.6)	0.39 (1.4)	0.44 (0.7)	0.87 (0.9)	0.45 (1.0)
MVPA (mins/day)	89.1 (13.7)	46.3 (6.2)	83.3 (14.2)	40.2 (12.5)	64.6 (25.0)
Sedentary time (mins/day)	427.3 (68.6)	472.5 (42.1)	466.2 (69.4)	471.1 (51.0)	459.2 (59.9)
**Parent characteristics**					
Gender (% female)	50.0%	36.4%	70.0%	55.0%	50.0%
Age (years)	44.0 (6.3)	42.6 (5.8)	44.1 (6.8)	39.6 (4.0)	42.5 (5.9)
BMI (kg/m2)	24.5 (3.0)	28.3 (7.4)	23.1 (2.7)	25.2 (4.2)	25.3 (4.9)
IMD	11.9 (5.6)	18.3 (18.1)	15.0 (11.0)	22.6 (18.6)	16.9 (14.5)
MVPA (mins/day)	66.9 (28.2)	53.2 (26.1)	70.3 (20.1)	46.9 (19.4)	58.9 (24.8)
Sedentary time (mins/day)	539.5 (44.6)	525.4 (47.4)	534.8 (82.9)	524.8 (61.8)	530.8 (58.9)
Ethnicity (% White British)	100.0%	100.0%	90.0%	90.9%	95.2%
Employment status					
Full time	40.0%	54.5%	70.0%	36.4%	50.0%
Part time	40.0%	27.2%	30.0%	63.6%	40.5%
Not in paid employment	20.0%	18.2%	0%	0%	9.5%

**Table 2:** Key findings and recommendations to support physical activity during holidays.

Finding	Recommendation
Grandparents are a key source of childcare during the school holidays	Support for grandparents in providing frequent physical activity opportunities are needed. Increasing grandparents’ own physical activity may also be effective.
Holiday clubs are not appealing to older children	Greater provision for older children (11+). Develop clubs in collaboration with this age group to ensure they are age appropriate and appealing.
Children would like to attend holiday clubs, but parents are unable to afford them	Better signposting for parents to more affordable optionsImprove the cost-effectiveness and value of current provision (e.g., by providing meals, additional benefits outside of being active)
The location of organised activities and facilities is a key barrier to parents providing opportunities for PA every day during the holidays	Ensure equity of provision of physical activity clubs and facilities in terms of location, with a particular focus on rural areas
